# Brazil's Yanomami health disaster: addressing the public health emergency requires advancing criminal accountability

**DOI:** 10.3389/fpubh.2023.1166167

**Published:** 2023-05-17

**Authors:** Erick Da Luz Scherf, Marcos Vinicius Viana da Silva

**Affiliations:** ^1^Faculty of Social Sciences, University of Stavanger, Stavanger, Norway; ^2^Universidade do Vale do Itajaí, Itajaí, Santa Catarina, Brazil

**Keywords:** Brazil, Yanomami, Indigenous health, public health emergencies, criminal accountability

## Introduction

In this opinion piece, we consider the recently publicized public health emergency affecting the Yanomami Indigenous people in Brazil. We suggest that tackling this crisis also requires a serious commitment toward accountability for those responsible for it.

### Disease and death among the Yanomami: contextualizing a foreseen crisis

On January 20th, 2023, around 3 weeks after the newly elected government headed by Lula da Silva took office in Brazil, the Brazilian Ministry of Health declared the situation of the Yanomami Indigenous people to be a “public health emergency of national relevance” due to the severe lack of health and social assistance to that population (1). After 4 years of destructive public health policies brought forward by Jair Bolsonaro's administration (2019–2022), which were largely driven by science denialism, necropolitics, and neoliberal authoritarianism (2), the biopsychosocial wellbeing of several vulnerable groups in the country, especially Indigenous communities, has been seriously impaired. Bolsonaro's government (2019–2022) has been characterized before as a neofascist regime, one that showcased a clear disregard for human rights, especially concerning the lives and rights of minorities. There is enough evidence in the case of the Yanomami health disaster that indicates that crimes against humanity, possibly including the crimes of genocide and ethnic cleansing were committed against these populations. Yet, so far, criminal responsibility has been minimal, which led to the development of this Opinion piece.

**Figure 1:**
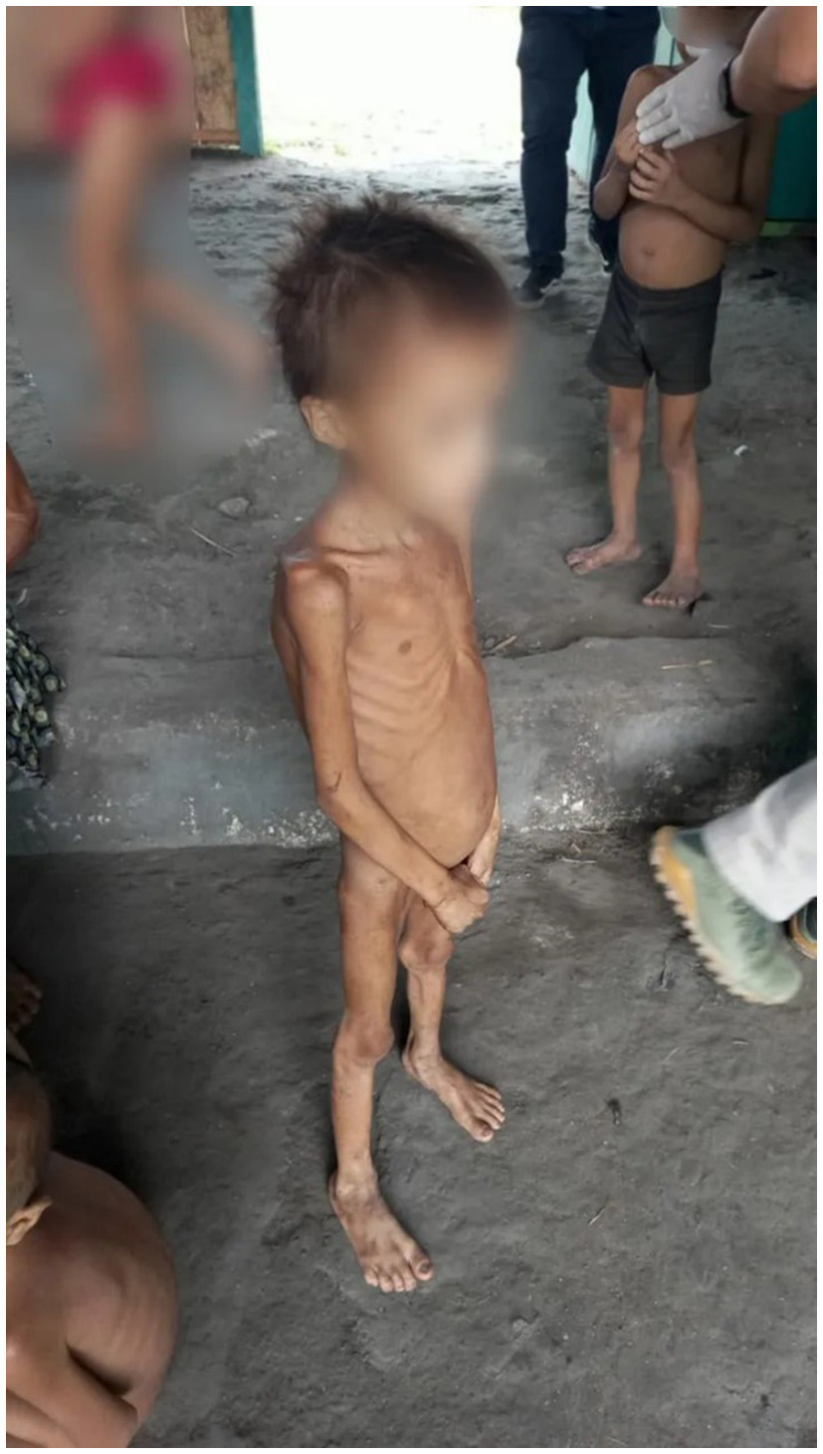
Yanomami child, visibly malnourished. Source and photo credit: SUMAÚMA, https://sumauma.com/en/nao-estamos-conseguindo-contar-os-corpos/.

During the COVID-19 outbreak, marginalized groups were treated as “expendable surplus” by the previous federal administration (3), and violence against Indigenous peoples has soared, especially in the Amazon Region (4). As a result of that, in addition to the unprecedented levels of deforestation, illegal mining, logging, poaching, and land grabbing in Indigenous territories (which increased, in general, by 137% under Bolsonaro's administration in 2020 alone) (5), public health and epidemiological indicators for Indigenous peoples in Brazil have considerably worsened (6), especially after the new coronavirus outbreak in early 2020 (7). These events led up to the death of at least 570 Yanomami children over 4 years, mainly due to curable diseases, including malnutrition (see Figure 1), malaria, and diarrhea, as well as malformations attributable to the mercury used by illegal gold miners (8).

We argue that to address this public health emergency, the crisis affecting the Yanomami people in Brazil right now should be viewed in light of the crimes committed against this population, crimes that build up on possible violations of human rights, including violations of the right to life, and the right to the highest attainable standard of health (2). We also briefly discuss the implications of judicialization in Brazil and its meaning for the future of Indigenous people's health and wellbeing.

The Federal Government under Bolsonaro's administration chose to completely ignore the situation affecting the Yanomami people in Northern Brazil. Throughout Bolsonaro's first and only term, there were at least 21 requests for help pertaining to the situation of the Yanomami people, all of which were ignored by the former President (9). In 2021, a local Civil Defense agency tried to alert Bolsonaro's Minister of Human Rights Damares Alves (now Senator-elect) of a possible food crisis and her Ministry did not take any concrete action to prevent it from escalating (10). Several antimalarial drugs sold by the Oswaldo Cruz Foundation to the Federal Government somehow ended up in the hands of illegal miners, who built an entire business around selling these life-saving medications in the Yanomami territory. Therefore, a myriad of governmental and non-governmental actors have contributed to this foreseen crisis in Brazil, which means that criminal accountability needs to take place across a wide range of sociopolitical actors.

### Indigenous health and territories under attack in Northern Brazil

**Figure 2:**
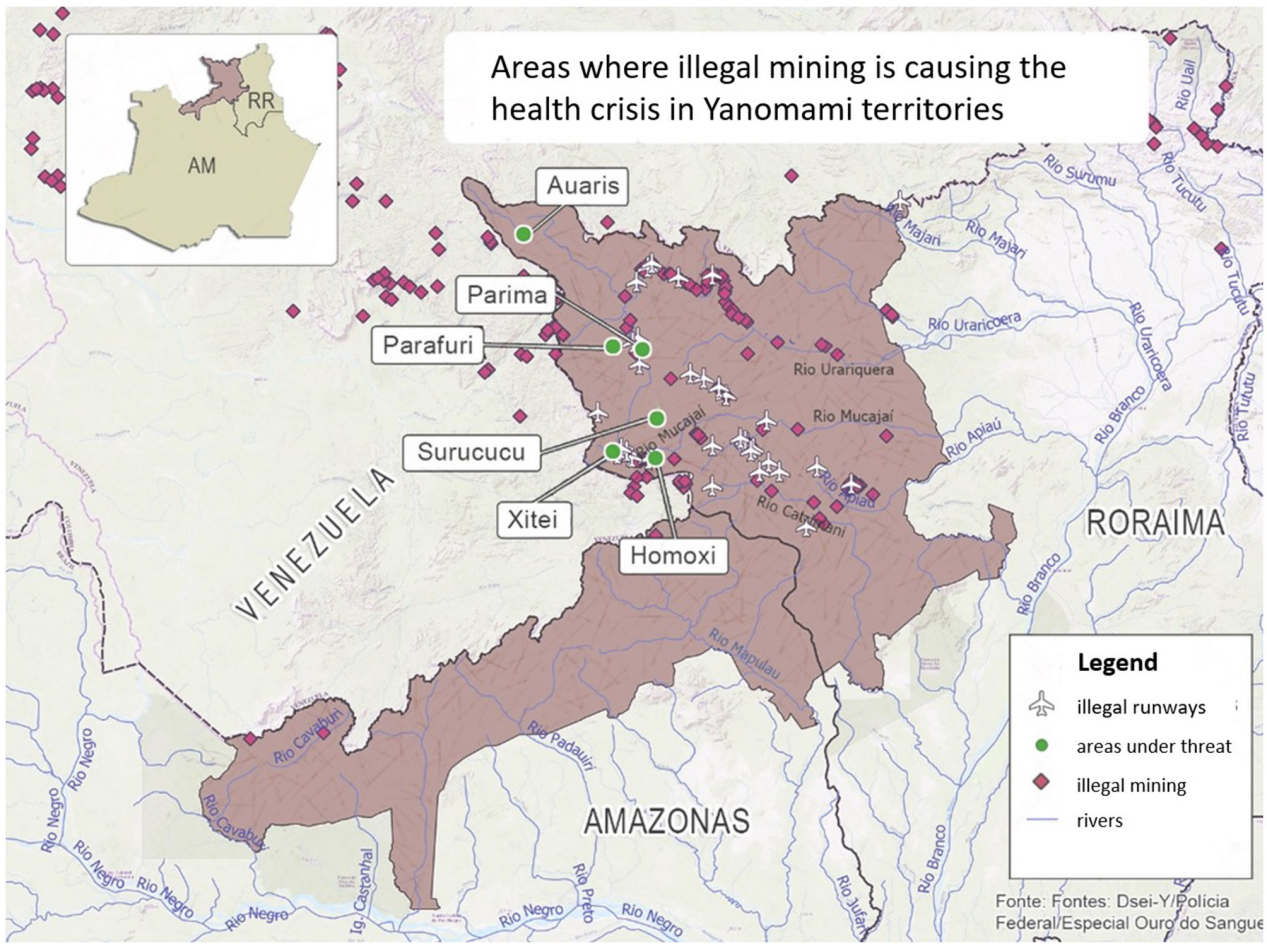
Map of territories inhabited by the Yanomami and where illegal mining usually takes place. Source: adapted from Amazonia Real, https://amazoniareal.com.br/criancas-yanomami-2/.

It is estimated that around 30,000 Yanomami people live across the states of Roraima and Amazonas in Northern Brazil, with some dwelling in South Venezuela as well, see Figure 2 (11). They are the largest Indigenous group in South America living in relative isolation. Although the Yanomami have struggled for their livelihood and territories for decades against several interventions from the Brazilian government, military soldiers, and gold miners (9), recent attacks on their lands, life, and rights have led to one of the worst public health emergencies affecting Indigenous people in Brazil (12).

The death of hundreds of Yanomami children, in addition to dozens of hospitalizations (12), poses a serious threat to the future of the Yanomami people and their very capability of existing in the long term. During the COVID-19 outbreak in Brazil, several Indigenous leaders and elders have died, partially because of Bolsonaro's administration and its lack of commitment to combating the virus or promoting Indigenous health (2, 13). Some say that when a tribal chief or elder dies, a whole library dies with them, considering the way knowledge is transmitted from generation to generation through oral traditions (13). By allowing Yanomami children to die from preventable diseases, Brazil is endangering the future of these communities, and by allowing the elders to perish, we are losing an important part of their past, history, and ancestrality.

Since the election of former president Jair Bolsonaro in 2018, environmental protection efforts and regulations, especially in the country's Amazon region, have been considerably diminished. To the point where illegal activities led by anti-environmental militias have grown at unprecedented levels, with consent from the federal government led by Bolsonaro (14). These activities involve land grabbing, illegal deforestation, and illegal mining (14) which is the most prejudicial one to Indigenous peoples in Northern Brazil, including the Yanomami. Even though the public health impacts of mining activities have been widely described before (15), the Brazilian case stands out for all the wrong reasons: as “the onslaught of illegal miners into Indigenous territory in the Brazilian Amazon has destroyed forest, polluted rivers, and brought disease and malnutrition to the Yanomami people” (16). With the advancement of Jair Bolsonaro's anti-environment crusade after his election, illegal deforestation caused by illegal mining activities (better known as *garimpo ilegal* in Brazilian Portuguese) increased by 309% between 2018 and 2022 (17). Farmers, land grabbers, gold market entrepreneurs, and illegal miners all felt encouraged by Bolsonaro to assault Indigenous people and their lands. Illegal activities in Indigenous territories were frequently encouraged by the former President himself, through lives on social media, interviews, and bills proposing the legalization of mining activities in Indigenous lands, which is strictly prohibited by the Constitution (18). In the case of the Munduruku, Kayapó, and Yanomami Indigenous lands, illegal mining had expanded by more than 300%, a clear example of how businesspeople (criminals in this case) have taken advantage of impunity, the high price of minerals and the ease, in times of misery, of recruiting poor and working-class people to help them line their pockets (18).

These events describe a systematic attempt from different sociopolitical actors in Brazil (who benefited from the acquiescence of Brazilian public authorities for the past 4 years), including farmers, land grabbers, illegal miners, and gold entrepreneurs in Brazil and abroad, to grow their businesses at the expense of Indigenous lives.

## Discussion

### Public health meets criminal law: the Yanomami crisis and the way forward

So far, the new federal administration led by President Lula has taken several actions to deal with the Yanomami health crisis head-on. Measures are being put in place to provide emergency medical and social assistance to Yanomami people as well as to stop more illegal activity from happening in their territories (8, 11). However, we argue that without a serious commitment to the responsibilization and accountability of those responsible for this crisis, the future of Indigenous health and rights can be seriously compromised.

Even though judicialization of these issues will not automatically lead to better public health outcomes for either Indigenous or non-Indigenous people, criminal law can and should be used to enforce public health regulations and protect public health. Criminal laws can be used to prosecute individuals and organizations that engage in behaviors that pose a risk to public health, especially in the case of illegal mining which has promoted mercury contamination and diseases of public health significance such as malaria, leishmaniasis, and others (16). In the midst of this crisis, there is an opportunity for Lula's administration to demonstrate its commitment to environmental protection and that it is ready to protect not only the Amazon rainforest but the people who lived there in harmony with nature for centuries before the invasion by these anti-environmental militias. After a large coverage by the media and recent actions from the federal government, illegal gold miners (better known as *garimpeiros*) were seen fleeing the Yanomami territory toward other regions in Northern Brazil, and accountability for their acts remains an open question.

After contaminating the water, spreading diseases of public health concern, and even raping Yanomami children (19), will the people responsible for these crimes be held accountable to the full extent of the law? What about the public authorities that chose to ignore it all? Are we, as a society, going to simply forgive and forget? Accountability is a crucial component of public health, therefore, in order to strengthen our public health system for the benefit of both Indigenous and non-Indigenous communities, mechanisms shall be set in motion to investigate and punish all sociopolitical actors responsible for the ongoing Yanomami health crisis.

## Concluding remarks

Brazil's Yanomami health disaster is one that was foreseen if not premeditated. Nonetheless, it is important to note that the almost 20,000 illegal miners who invaded the Yanomami land and contributed to the deepening of this health crisis are just one piece of the puzzle. Not taking anything away from their responsibilities, these miners are often poor/working-class people who saw an opportunity to make quick cash and jumped at it. Yet, there is a wide range of other actors who contributed directly or indirectly to the crimes against the Yanomami and other Indigenous populations. These include several government agents part of the former federal administration led by Jair Bolsonaro, state, and municipal authorities, in addition to gold sellers and companies in Brazil and even in the United States (including famous Big Tech customers) who benefited from minerals extraction, purchase, and sales originating from illegal activities in Indigenous lands (20). With that said, public authorities, including the Federal Public Prosecutor's Office (Ministerio Publico Federal), Congress, and Civil Society actors should demand that criminal responsibility be extended to all those involved in the making of this crisis while respecting the principles of individuality concerning their degree of involvement, as without this the public health emergency cannot be completely remediated.

## Author contributions

ED was responsible for the study design, most of the writing, spell-checking, and the final layout. MV was mainly responsible for the data collection, gathering the sources, writing up, and revising the text. Both authors contributed equally to the final outcome. Both authors contributed to the article and approved the submitted version.
